# Modeling Possible Outcomes of Updated Daily Values on Nutrient Intakes of the United States Adult Population

**DOI:** 10.3390/nu12010210

**Published:** 2020-01-13

**Authors:** Jill C. Newman, Michael I. McBurney, Kelly J. Hunt, Angela M. Malek, Bernadette P. Marriott

**Affiliations:** 1 Department of Medicine, Medical University of South Carolina, Charleston, SC 29425, USA; newmanji@musc.edu; 2 Human Health & Nutritional Sciences, University of Guelph, Guelph, ON N1G 2W1, Canada; mcburne@uoguelph.ca; 3 Department of Public Health Sciences, Medical University of South Carolina, Charleston, SC 29425, USA; huntke@musc.edu (K.J.H.); malek@musc.edu (A.M.M.); 4 Department of Psychiatry and Behavioral Sciences, Medical University of South Carolina, Charleston, SC 29425, USA

**Keywords:** Daily Values (DV), Nutrition Facts Label, Estimated Average Requirement (EAR), Upper Limit (UL), usual intake (UI), fortification, adults, National Health and Nutrition Examination Survey (NHANES)

## Abstract

The United States (US) Food and Drug Administration has updated the Daily Values (DVs) for the Nutrition Facts Label on packaged foods. We used the National Health and Nutrition Examination Survey 2009–2012 data with the International Life Sciences Institute, North America Fortification Database, which identifies intrinsic, mandatory enriched, and fortified sources of nutrients in foods and beverages, to model the new DVs’ potential impact on adult (≥19 years of age) intake. We assumed that manufacturers will adjust voluntary fortification to maintain percent DV claims. We assessed the percent of the US population whose usual intake (UI) was < the Estimated Average Requirement (EAR), and ≥ the Upper Limit (UL) based on the current DVs, and modeled estimated UI and %<EAR with the new DVs (Updated DV) for 12 micronutrients. Modeling for vitamins B_12_, A, B_6_, riboflavin, niacin, thiamin, and zinc predicts fewer voluntarily fortified foods and reduced adult UI. Assuming manufacturers add more vitamins C and D and calcium to foods, the Updated DV predicts the adult UI will increase for these nutrients. Our modeling predicts a 15% reduction in overall adult vitamin A intake, a recognized “shortfall nutrient” and that even with the increased DV for vitamin D, 70% of US adults are predicted to have an intake <EAR.

## 1. Introduction

Nutrients added through enrichment and voluntary fortification of foods make important contributions to dietary intake in the United States (US) [1,2,3,4,5,6]. However, most Americans don’t consume recommended amounts of dietary fiber and what have been termed “shortfall nutrients” including vitamins A, C, D, and E, folate, magnesium, potassium, and calcium [7,8,9]. In addition, US men and women in the lowest income groups are more likely to experience inadequate intakes of these nutrients compared with the highest income groups [10].

Use of food labels by US consumers has been positively associated with dietary quality [11]. Specifically, regulated nutrition labels on packaged foods and beverages enable consumers to compare the nutrient contribution of standardized food serving sizes, which the US Food and Drug Administration (FDA) has defined as the Reference Amounts Customarily Consumed (RACCs) (FDA 21 Code of Federal Regulations 101.12) [12]. The RACCs on food and beverage products are used to generate the “% Daily Values” (%DV) on the Nutrition Facts Label, which help consumers ‘know how much a serving contributes to the *total amount you need* per day’ [13].

On 20 May 2016, the FDA announced a new Nutrition Facts Label for packaged foods [14]. Manufacturers with ≥$10 million and <$10 million in annual food sales will be required to display the new labels beginning 1 January 2020 and 1 January 2021, respectively. As part of the new Nutrition Facts label changes, the FDA updated the DVs for most vitamins and minerals with more recent reference values based on the newer Food and Nutrition Board (FNB) values and recommendations [15,16]. Specifically, the FDA has increased the DVs for three vitamins and five minerals, decreased the DVs for 14 vitamins and minerals, and did not change the DVs for three nutrients, with the impact on the DVs for two vitamins dependent upon their chemical structure [17]. 

The DVs were established in 1995 based on values in the FNB, National Academies of Sciences Engineering and Medicine report, Recommended Dietary Allowances (RDAs) [18], and an FDA-commissioned FNB consensus document on nutrition labeling [19]. From 1994 to 2005, the FNB revised and broadened the national nutrient intake recommendations as the Dietary Reference Intakes (DRIs), which include RDA values, and have been periodically updated since that time cf. [20]. 

In the US, the DVs are also used by food and supplement manufacturers when making nutrient content claims. A food product can be labeled as “high” or “excellent” for a nutrient if it contains ≥20% DV per RACC—a “good” source of the nutrient if it contains 10–19% DV per RACC. As manufacturers update Nutrition and DS Facts labels to meet 2020 or 2021 compliance dates and DVs, they will need to update product %DV claims. To adhere to FNB recommendations and align product claims with updated DVs, manufacturers may decide to reformulate fortified products [17].

The objective of this analysis was to examine the potential impact of the updated DVs on voluntary fortification of food products and usual nutrient intakes. We used dietary intake data from the National Health and Nutrition Examination Survey (NHANES) coupled with the International Life Sciences Institute, North America Fortification Database (ILSI NA-FD) [21], which contains information on the intrinsic, enriched, and voluntary nutrient levels of vitamins A, B_1_ (thiamin), B_2_ (riboflavin), B_3_ (niacin), B_6_ (pyridoxine), B_12_ (cobalamin), C, D, folate as Dietary Folate Equivalents (DFEs), calcium, iron, and zinc in foods. For this analysis, the ILSI NA-FD was replicated and the fortified nutrient contents of foods were adjusted to fulfill the updated labeling and DV requirements, while maintaining the same %DV in each fortified food.

## 2. Materials and Methods

### 2.1. Study Population and Dietary Intake Estimation

Dietary intake information reported for two consecutive NHANES cycles (2009–2010, 2011–2012) [22] was combined with the US Department of Agriculture (USDA) food patterns equivalents database and with the ILSI NA-FD [21]. The NHANES is a nationally representative, cross-sectional survey that samples noninstitutionalized, civilian US residents using a complex, stratified, multistage probability cluster sampling design and is collected by the National Center for Health Statistics (NCHS) of the Centers for Disease Control and Prevention (CDC) on a continual basis [23].

The NHANES dietary data include two 24-h dietary recalls collected using a computer-assisted dietary interview software program: the USDA’s automated multiple-pass method. The first 24-h dietary recall is conducted as an in-person interview and the second is administered by telephone 3–10 days later. Food and beverage items reported consumed in the NHANES dietary interview component (What We Eat in America) were coded using the USDA’s Food and Nutrient Database for Dietary Studies (FNDDS) and their estimated nutrient content was obtained [24].

Although complete 24-h dietary intake data were available for 18,273 participants on Day 1 and Day 2 from the NHANES (2009–2010, 2011–2012) combined data, after exclusion of participants whose data were deemed by the interviewer to be incomplete (*n* = 261), participants who were <4 years of age (n = 1991), and women who were pregnant or lactating (*n* = 168), the sample included a total of 15,853 participants. The sample was further reduced to only include adults ≥19 years of age to produce an analytic sample of 10,698 participants in this study. In this total sample, 50.0% were male.

We also used the ILSI NA-FD (Database of Fortification, Enrichment, and Intrinsic Nutrient Levels in Foods Reported Consumed in What We Eat in America, NHANES 2009–2010 and 2011–12, version 1.0, completed 16 October 2015) [21], which contains estimates of nutrients available from three sources—as naturally occurring (i.e., intrinsic), enriched, and fortified—for those foods and beverages as reported in the two NHANES cycles (2009–2010 and 2011–2012). The ILSI NA-FD is based on the USDA’s FNDDS, and the original database was developed by Nutrition Impact LLC, Battle Creek, MI, USA and has been updated by Exponent, Inc. (Menlo Park, CA, USA). Some values for folate content of foods in the ILSI NA-FD were sourced from the USDA FNDDS (release 28; released September 2015, slightly revised May 2016).

### 2.2. Usual Intake Estimation

Data obtained from the two NHANES 24-h recalls were used to estimate prevalence of intake following the US National Cancer Institute (NCI) usual intake (UI) estimation methodology [25,26]. The NCI’s MIXTRAN and DISTRIB computer macros enable UI estimation at the individual level. As part of our analysis, we incorporated/controlled for age, interview day (first compared with second), and weekend day (yes/no) to account for weekend effects in intake.

Estimated UI data were generated from: (1) foods and beverages only as naturally occurring (intrinsic), (2) foods and beverages including those with enrichment, and (3) all foods and beverages including voluntary fortification for vitamins A, B_1_ (thiamin), B_2_ (riboflavin), B_3_ (niacin), B_6_ (pyridoxine), B_12_ (cobalamin), C, D, folate, calcium, iron, and zinc. DFEs were used for folic acid and folate [16].

### 2.3. Creation of the Current DV and Updated DV Datasets

First, we replicated the ILSI NA-FD (Original FD) and created a second database ILSI NA-FD (Modified FD) in which voluntary fortification levels were adjusted to maintain the same %DV in foods reported in the Original FD but applying updated DVs that will be effective in 2020 or 2021 (see Table 1). In creating the Modified FD, three assumptions were made: (1) the naturally occurring intrinsic nutrient content of a food remained constant; (2) the amount of nutrients added to a food through enrichment was held constant; and (3) food manufacturers will maintain the same %DV in food products after 2020 or 2021. Appendix A Table A1 provides details about the calculations used in creating the ILSI NA-FD Modified.

An enriched food is a product to which nutrients have been added that were typically present in the food in its original form but were lost during processing. Food enrichment in the US is expected to follow the FDA’s food restoration principle, cf. [27]. In the US, food fortification is governed by the FDA and refers to the practice of increasing the content of micronutrients in a food to improve its nutritional quality [27]. In this study, we assumed that the amount of nutrients added through enrichment would be held constant because the US regulations specifying the amounts of vitamins A, thiamin, riboflavin, niacin, D, folic acid, and iron to be added when enriching foods were not altered when the DVs were updated [12]. In addition, the amount of nutrients added during enrichment are often linked to standards of identity in the US [17]. A standard of identify establishes the name of the food, e.g., yogurt, and defines the ingredients or components, their function and levels permitted in food (21 Code of Federal Regulations 130.3) [28] or foods that substitute (21 CFR 130.10) [29].

When the intrinsic and enrichment levels of a nutrient in a food would yield the same (or greater) %DV in the Modified FD as in the Original FD, we also assumed food manufacturers would discontinue voluntary fortification. Therefore, in our model, we set the fortification contribution to zero and categorized the food as non-fortified in the Modified FD. This condition was the only one where we reassigned a food/beverage from fortified in the Original FD to non-fortified in the Modified FD. Since the Original FD reports DFE for folate/folic acid, and does not report whether a vitamin E fortified food contains µg of synthetic or natural vitamin E, nutrient levels were left unchanged for these two nutrients when creating the Modified FD.

Second, the NHANES individual food intake files were merged with the Original FD, which contained the total amount of each nutrient in the specified food/beverage and proportions according to the three sources (naturally occurring, enriched, and fortified). The total amount of each nutrient in food and source type (naturally occurring, enriched, fortified) for the analysis was calculated by multiplying the number of grams per nutrient from the NHANES food file by the nutrient proportion in the ILSI NA-FD, then dividing by 100. This yielded a total nutrient intake from all foods being consumed and the proportion that was intrinsic, enriched or fortified for each individual. The same modeling approach was used with the Modified FD.

Third, nutrient by nutrient and using the DV relevant to each model, individuals were categorized according to the proportion of their nutrient intake derived from fortified foods as follows: (a) 0% of DV consumed in the form of fortified food (0% DV), (b) >0–50% of the DV in the form of fortified food (>0–50% DV), or (c) >50% of DV in the form of fortified food (>50% DV).

Fourth, using the two 24-h dietary intake recalls per person, Current DV and Updated DV models were run independently using the Original and Modified FDs, respectively, to obtain usual nutrient intake for each individual and nutrient distribution. In addition, mean UI, mean percentage below the Estimated Average Requirement (% <EAR), and mean percentage greater than or equal to the Tolerable Upper Limit (% ≥UL) were determined for the population in both models.

### 2.4. Statistical Analysis

We accounted for the NHANES clustered sampling design and oversampling in all analyses and adjusted for differential noncoverage and nonresponse across the two continuous NHANES cycles [30,31,32]. Frequencies were reported for sample size, number of foods that were fortified and enriched, and number of foods with ‘Good’ or ‘Excellent’ source nutrient content claims. Means and standard errors of the mean (SEM) were calculated for average UI, percentage below the EAR (% <EAR), and percentage greater than or equal to the UL (% ≥UL). SEs were estimated using Balanced Repeated Replication and NHANES weights were applied. The percent of nutrient intake derived in the form of fortified food was calculated by dividing the total fortified nutrient intake consumed by the DV times 100. We analyzed the data for all adults ≥19 years of age for both sexes combined. All analyses were conducted using SAS version 9.4 and its complex survey-specific procedures (SAS Institute, Cary, NC USA). The NHANES survey protocol has been approved by the NCHS Research Ethics Review Board. Since this study was a secondary data analysis of publicly available federal data, Human Subject Institutional Review Board approval was not required by the Medical University of South Carolina.

## 3. Results

With the new FDA labeling rules, the DV was reduced for seven nutrients (vitamins A, thiamin, riboflavin, niacin, B_6_, B_12_, and zinc) and increased for vitamins C and D, and calcium (Table 1). In general, foods in the US are most often fortified with vitamins A and D and enriched with riboflavin, niacin, thiamin, folate, and iron (Table 2). In our modeling, voluntary fortification was adjusted in the Modified FD to achieve the same numerical %DV for each nutrient and food as found in the Original FD. For the seven nutrients where the DV was reduced, the number of foods predicted to be voluntarily fortified decreased for all except vitamin B_6_ (Table 2). These voluntarily fortified foods (Modified FD) would contain less of these seven nutrients per serving or RACC compared to the Original FD. When a nutrient DV is not changed, the number of fortified foods does not change, as was the case for vitamin E, folate DFE, and iron. When a nutrient Updated DV is higher, such as for vitamins C and D, and calcium, the amount of nutrient added to a voluntarily fortified food was increased in the Modified FD, whereas the amount found intrinsically in foods or added via enrichment was unchanged.

The decisions by food manufacturers to voluntarily add more nutrients per serving to maintain the same %DV in a fortified food or to fortify new foods (vs. Original) will depend upon palatability and may require regulatory changes to ‘standards of identity’ to permit the addition of more vitamins or minerals (21 Code of Federal Regulations 130.3) [28]. In our modeling, we discounted consideration of palatability or standards of identity limitations, to estimate the number of foods that would be predicted to carry ‘Good’ and ‘Excellent’ source claims. As shown in Table 3, we found predicted increases for the B vitamins, vitamin A, and zinc for ‘Good’ and ‘Excellent’ claims, even though nutrient density is unchanged (intrinsic and/or enrichment foods) or diminished through voluntary fortification. Presuming manufacturers add calcium and vitamins C and D to fortified foods to maintain the same %DV, the number of ‘Good’ and ‘Excellent’ source foods is reduced. This reduction occurs because the intrinsic content of a food or the enrichment amount allowed by ‘standards of identity’ regulations is insufficient to maintain the higher nutrient content claim. For example, US regulations (Code of Federal Regulations 131.110) [33] permit the addition of 400 International Units (IU) of vitamin D per quart (400 IU or 10 μg per 32 fluid ounces) of cow’s milk [17]. With the Updated DV (20 μg) being twice the Current DV (400 IU), an eight-fluid ounce glass of milk containing 100 IU (2.4 μg) vitamin D will be downgraded from an ‘Excellent’ source (25% Current DV) to a ‘Good’ source (12.5% Updated DV) of vitamin D.

Table 4 shows that the Updated DV model predicts a small change in the number of adults aged ≥19 years consuming foods that are not voluntarily fortified with the exception of vitamin A and to a lesser extent, zinc. However, the Updated DV model predicts that more individuals should be obtaining >50% of the DV for vitamin D from fortified foods, i.e., more than 10 µg per day. The Updated DV model predicts an appreciable drop in the number of individuals obtaining vitamin A from fortified foods, i.e., >50% and >0–50% DV categories for vitamin A. This downward shift in consumption of fortified foods is exacerbated given the lower DV target in the Updated DV than the Current DV model. The Updated DV model finds a small increase in the number of people not consuming foods voluntarily fortified with B vitamins, except for niacin, vitamin B_6_, and folic acid, and a large increase (40%) in the number of people not consuming foods fortified with vitamin A—a “shortfall nutrient” of national concern [8]. The number of foods voluntarily fortified with calcium, vitamin C, and vitamin D was held constant in the model; predictably, the number of people consuming these fortified foods will not change.

Table 5 includes the UIs and the percent of individuals in the US with intakes below the EAR and above or equal to the UL (as available) for the ten modeled nutrients. Since the DVs were unchanged for vitamin E, folate and iron, UIs did not differ between the two models and these nutrients were excluded from these analyses. Comparing the two models with the Updated DV, the overall mean UI of vitamin B_12_ and vitamin A is predicted to decrease, whereas the mean UI for vitamin C and vitamin D (almost double) and calcium is predicted to increase. The mean UI for thiamin, riboflavin, niacin, vitamin B_6_, and zinc is predicted to remain relatively unchanged. The model predicts a reduction in %<EAR for vitamins C and D and calcium along with an increase in the percentage of the population exceeding the UL for calcium. Despite a doubling of DV for vitamin D and an assumption that food manufacturers reformulate fortified food products to maintain the same %DV, approximately 70% of adults still are predicted to have a vitamin D intake <EAR. For all seven nutrients with a decreased DV, there is an increase in the percentage of the population below the EAR (Table 5). With the change in DVs, the only predicted decrease in the percentage of the population consuming more than or equal to the UL is for niacin.

Appendix BTable A2, Table A3, Table A4 and Table A5 which provide comparison modeling data on adult age group subsets: 19–30 years; 31–50 years; 51–70 years; >70 years are available in Appendix B. Within these age groups, the same trends were observed as with the overall adult intakes with the Current DV and Updated DV models. However, comparisons of adults aged 19–30 years (vs. all ages reported in Table 5) predict an increased proportion of this age group would have nutrient intakes below the EAR, especially for vitamin A, with the exception of vitamin B_6_ and calcium. Based on the %<EAR, adults aged 31–50 years are predicted to be generally better nourished than the overall population, whereas proportionally more adults aged 51–70 years were estimated to have intakes <EAR for vitamin B_6_ and ≥UL for calcium. Compared with the overall adult population shown in Table 5, a lower percentage of adults aged >70 years would be expected to have intakes <EAR for vitamins A, C and D and more individuals of this age group would be expected to ingest <EAR for the B vitamins. 

## 4. Discussion

Food availability and personal food choices affect dietary nutrient density and nutrient intake. When nutrient labeling requirements, such as the DV and targets for ‘Good’ and ‘Excellent’ nutrient claims are changed, the nutrient density of many food choices also may be affected [17]. The goal of this study was to determine the potential impact of regulatory application of newer FNB nutrient recommendations and codification as Updated DVs used in nutrition labeling on voluntary food fortification and the UI of select essential nutrients among American adults. The DV was decreased for 14 nutrients and increased for eight [17]. Using the ILSI NA-FD and NHANES datasets, we modeled 13 nutrients. Our Updated DV model predicts a reduction in the number of voluntarily fortified foods for six of the seven nutrients with a decreased DV, the exception being vitamin B_6_.

As part of this modeling approach, we did not transform a non-fortified food to a fortified food. Thus, the number of voluntarily fortified foods in the Modified FD was not altered for nutrients where the DV was increased (vitamins C and D and calcium), the DV was held constant (folate, iron), or the chemical form of the vitamin being added could not be determined (vitamin E and folate). However, for nutrients where the Updated DV is lower than the Current DV, food manufacturers have a choice to reduce voluntary fortification and maintain the same %DV claim on a food product. The Updated DV model predicts that a lowering of the DV may lead to a ~40% reduction in the number of foods voluntarily fortified with vitamin A and an increased number of foods meeting ‘Good’ and ‘Excellent’ nutrient content claims with a concomitant lower nutrient density.

The consumption of enriched and fortified foods is associated with increased nutrient intake and only a small percentage of US adults having total UIs below the EAR for vitamins B_6_ and B_12_, thiamin, riboflavin, niacin, folate, iron, copper, and selenium [2,3,6]. Based on this analysis, mandatory food enrichment, not voluntary food fortification, is primarily responsible for UI above the EAR for thiamin, riboflavin, niacin, folate, and iron. To benefit the US population, both enriched and fortified foods need to be available in the marketplace and then selected by the consumer for consumption. For nutrients where the DV increased, e.g., vitamins C and D and calcium, and assuming food manufacturers increase voluntary fortification to maintain the same %DV, these fortified foods should become more nutrient dense. However, it is worth noting that the intrinsic and enriched contribution of these nutrients will not be sufficient for foods which currently carry ‘Good’ or ‘Excellent’ claims to continue to do so under the Updated DV regulations. There appears to be an opportunity to modify US regulations pertaining to standards of identity and enrichment of foods to allow the addition of more vitamins and minerals and align with updated DVs.

Limitations to our analysis include that dietary intakes based on 24-h dietary recalls are subject to misreporting; however, the newer multiple-pass method of dietary interviewing used to collect the data for the NHANES cycles included in this modeling has been shown to be much improved and significantly reduces bias [37,38,39]. Another limitation is that the vitamin E and folate/folic acid content of foods in the database could not be verified because it is not possible to ascertain the chemical structure added to foods or the conversion factor used for the label. This is unfortunate given the low intake of vitamin E among the US population [2,3,6], and the importance of folic acid in fetal development [40,41]. In addition, the food database used does not capture every food in the marketplace and food manufacturers may make different voluntary fortification adjustments. The analysis also does not evaluate the impact on consumer food choices when comparing food labels with new DVs and related nutrient content, e.g., ‘good’ source, claims. Hopefully, results from this analysis may help inform food manufacturers in product development and help identify additional opportunities, e.g., regulatory changes to standards of identity and levels of enrichment, to improve the nutrient intake of US adults.

There are several strengths to this analysis. The estimates are nationally representative of the US population and based on the ILSI NA-FD that reports the intrinsic, enriched (mandatory) and voluntary fortification of each food serving (Original FD). The nutrient database was replicated, new DVs were applied, and the need for voluntary fortification was determined for each nutrient in every food (Modified FD). This created two databases that were used to estimate two sets of nutrient intakes (Current DV and Updated DV) using the same dietary recalls and statistical methods. This means that the UIs, EARs, and ULs are identical in 24-h dietary recall data and foods within the database.

## 5. Conclusions

This analysis highlights the fact that changing the DV may affect the nutrient density of foods and nutrient content claims. When the DV increases, it may lead to a downgraded nutrient content claim, i.e., from an ‘excellent’ to a ‘good’ source or from a ‘good’ source to no claim, unless voluntary fortification is increased. When the DV is decreased, the number of foods eligible to carry ‘Good’ and ‘Excellent’ nutrient content claims increase without any change in nutrient density. There may also be an ~40% reduction in the number of foods voluntarily fortified with vitamin A. Such changes in nutrient density of foods affect UI and % below the EAR.

The Updated DV model predicts that more people will not consume foods voluntarily fortified with vitamins A, thiamin, riboflavin niacin, B_6_, B_12_, and zinc. Our modeling also predicts that there will be a decrease in the proportion of the population obtaining up to 50% or >50% of their nutrient DV by means of voluntarily fortification. This translates to lower UI in the Updated DV Model for vitamins A, niacin B_12_, and zinc, but not thiamin, riboflavin, and vitamin B_6_. The greatest reduction in UI is predicted for vitamin A. This analysis found that 75% of adults currently consume foods fortified with vitamin A, and the Updated DV model predicts this will decrease to 65%, which is troubling because vitamin A has been recognized as a nutrient already deemed of national public health concern by the 2015 Dietary Guidelines Advisory Committee [8]. Separate adult subgroup age analysis predicted that the highest proportion of individuals with intakes < EAR for vitamin A would be individuals age 19–30 year. Even with a significant increase in the DV for vitamin D, our modeling still predicts that approximately 70% of the US adult population will have a UI < the EAR for this nutrient. A number of studies have demonstrated the value of fortification and enrichment in the US food supply [1,3]. As the US continues to address the related issues of food deserts, obesity, and shortfall nutrients [8,42,43], it is disconcerting to find that approximately 70% of adults also report not consuming any fortified foods.

## Figures and Tables

**Table 1:** Current and updated Daily Values (DVs) for food labels for adults aged ≥19 years ^1^ in the US population.

Nutrient	Current DV	Updated DV	Change, %
Vitamin B_12_	6.0 µg	2.4 µg	−60
Vitamin A	5000 IU	900 µg RAE ^2^	−40
Zinc	15 µg	11 µg	−27
Riboflavin	1.7 mg	1.3 mg	−24
Niacin	20 mg NE	16 mg NE ^3^	−20
Thiamin	1.5 mg	1.2 mg	−20
Vitamin B_6_	2.0 mg	1.7 mg	−15
Vitamin E	30 IU	15 mg ^4^	0
Folate	400 µg	400 µg DFE ^5^	0
Iron	18 mg	18 mg	0
Calcium	1000 mg	1300 mg	30
Vitamin C	60 mg	90 mg	50
Vitamin D	400 IU	20 µg ^6^	100

^1^ SOURCE: FDA 2018b [12]. DFE, Dietary Folate Equivalent; DV, Daily Value; IU, International Unit; NE, Niacin Equivalent; RAE, Retinal Activity Equivalent; US, United States. ^2^ 1 µg RAE = 1 µg retinol = 12 µg supplemental β-carotene = 24 µg α-carotene = 24 µg β-cryptoxanthin. ^3^ 1 mg NE = 1 mg niacin = 60 mg tryptophan. ^4^ 1 mg a-tocopherol (label claim) = 1 mg a-tocopherol = 1 mg RRR –a-tocopherol=2 mg all-rac-a-tocopherol. ^5^ 1 µg DFE = 1 µg naturally occurring folate = 0.6 µg folic acid. ^6^ 1 µg vitamin D = 40 IU.

**Table 2:** Number of foods that are fortified and enriched with specific nutrients in the Original Food Database and predicted in the Modified Food Database, NHANES 2009–2010 and 2011–2012 for the US adult population aged ≥19 years ^1^.

	2009–2010 Food Database			2011–2012 Food Database		
Nutrient	Fortified Original	Fortified Modified	Enriched	Fortified Original	Fortified Modified	Enriched
Vitamin B_12_	196	191	0	210	208	0
Vitamin A	1162	727	0	1153	652	0
Zinc	166	154	0	188	176	0
Riboflavin	221	219	1471	235	230	1403
Niacin	233	230	1594	248	247	1541
Thiamin	220	219	1600	238	235	1539
Vitamin B_6_	231	231	0	260	260	0
Vitamin E	75	75	0	74	74	0
Folate DFE	200	200	1593	220	220	1636
Iron	214	214	1576	232	232	1520
Calcium	196	196	7	204	204	6
Vitamin C	183	183	0	196	196	0
Vitamin D	855	855	0	854	854	0

^1^ Total of 4981 foods in the Original and Modified food database for 2009–2010 and 5192 foods in the Original and Modified food database for 2011–2012. Foods categorized as fortified may also be included in the enriched count. NHANES, National Health and Nutrition Examination Survey; US, United States.

**Table 3:** Number of Foods with Good or Excellent Source Nutrient Content Claims in the Original Food Database (Original FD), and the Predicted Number in the Modified Food Database (Modified FD), based on the NHANES 2009–2010 and 2011–2012 ^1^.

	2009–2010				2011–2012			
	Good Source		Excellent Source		Good Source		Excellent Source	
	Original	Modified	Original	Modified	Original	Modified	Original	Modified
Vitamin B_12_	1025	2101	579	1189	1150	2251	633	1326
Vitamin A	267	318	350	460	280	365	342	474
Zinc	670	880	482	721	682	911	466	691
Riboflavin	1284	1451	494	849	1318	1452	526	906
Niacin	974	960	915	1202	989	957	969	1266
Thiamin	756	872	765	938	829	958	762	940
Vitamin B_6_	844	1025	480	591	910	1093	547	673
Vitamin E	420	420	223	223	484	484	245	245
Folate ^2^	839	839	845	845	865	865	843	843
Iron	887	887	434	434	869	869	437	437
Calcium	752	516	334	229	794	504	332	236
Vitamin C	481	389	717	478	546	422	764	513
Vitamin D	357	268	239	200	404	296	291	243

^1^ Total of 4981 foods in the Original and Modified food database for 2009–2010 and 5192 foods in the Original and Modified food database for 2011–2012, NHANES, National Health and Nutrition Examination Survey. ^2^ DFE, Dietary Folate Equivalent.

**Table 4:** Number of US adults aged ≥19 years currently obtaining or predicted to obtain 0%, >0–50%, or >50% of the Daily Value (DV) from voluntarily fortified foods in Current and Updated DV models, respectively.

Nutrient	Current DV Model				Updated DV Model			
	DV	0%	>0–50%	>50%	DV	0%	>0–50%	>50%
Vitamin B_12_	6.0 mg	10,831	3267	1755	1.7 mg	10,869	3259	1725
Vitamin A	5000 IU	3644	11,687	522	900 μg RAE	5061	10,395	397
Zinc	15 µg	12,066	3364	423	11 µg	12,140	3313	400
Riboflavin	1.7 mg	10,550	4099	1204	1.3 mg	10,553	4128	1172
Niacin	20 mg	10,283	4141	1429	16 mg NE	10,292	4179	1382
Thiamin	1.5 mg	10,513	4222	1118	1.2 mg	10,522	4295	1036
Vitamin B_6_	2.0 mg	10,199	3909	1745	1.7 mg	10,199	3949	1705
Vitamin E	30 IU	14,349	1150	354	15 mg	14,349	1150	354
Folate	400 µg	11,083	1622	3148	400 µg DFE	11,083	1622	3148
Iron	18 mg	10,734	3375	1744	18 mg	10,734	3375	1744
Calcium	1000 mg	11,297	4049	507	1300 mg	11,297	4027	529
Vitamin C	60 mg	9726	2960	3167	90 mg	9726	2934	3193
Vitamin D	400 IU	3014	8952	3887	20 µg	2891	8686	4276

US, United States; DV, Daily Value; IU, International Unit; RAE, Retinal Activity Equivalents; NE, Niacin Equivalents; DFEs, Dietary Folate Equivalents.

**Table 5:** Comparison of Current Daily Value (DV) and Updated DV nutrient intake models with overall and percent fortification subgroups for US adults ≥ 19 years of age, (Mean ± SEM) ^1,2^.

	Current DV ^3^				Updated DV ^3^			
Nutrient	Overall	Percent Fortification ^4^			Overall	Percent Fortification ^4^		
		0%	>0–50%	>50%		0%	>0–50%	>50%
**Vitamin B_12_^5,10^**								
Sample size	10,677	7863	1674	1140	10,677	7883	1668	1126
Usual Intake	5.3 ± 0.0	5.3 ± 0.0	5.2 ± 0.0	5.3 ± 0.0	4.6 ± 0.0	4.6 ± 0.0	4.6 ± 0.0	4.6 ± 0.0
% < EAR	2.8 ± 0.4	2.8 ± 0.4	2.9 ± 0.4	2.8 ± 0.4	3.6 ± 0.4	3.5 ± 0.4	3.8 ± 0.6	3.6 ± 0.6
**Vitamin A ^6,9^**								
Sample size	10,681	2661	7681	339	10,681	3747	6664	270
Usual Intake	651.1 ± 0.2	641.4 ± 0.6	654.4 ± 0.4	653.4 ± 1.6	552.8 ± 0.2	547.5 ± 0.4	555.8 ± 0.2	553.1 ± 1.4
% < EAR	42.7 ± 2.2	44.9 ± 2.2	41.8 ± 2.0	45.3 ± 2.2	57.2 ± 2.4	58.8 ± 2.4	56.1 ± 2.4	60.3 ± 2.0
**Zinc ^6,9^**								
Sample size	10,697	8666	1773	258	10,697	8716	1738	243
Usual Intake	11.6 ± 0.0	11.6 ± 0.0	11.5 ± 0.0	11.5 ± 0.0	11.3 ± 0.0	11.3 ± 0.0	11.2 ± 0.0	11.2 ± 0.0
% < EAR	19.4 ± 1.0	19.3 ± 1.0	19.6 ± 1.0	22.5 ± 1.2	20.7 ± 1.0	20.5 ± 1.0	21.0 ± 1.0	24.0 ± 1.2
**Riboflavin ^5,10^**								
Sample size	10,697	7744	2187	766	10,697	7746	2201	750
Usual Intake	2.1 ± 0.0	2.1 ± 0.0	2.1 ± 0.0	2.1 ± 0.0	2.1 ± 0.0	2.1 ± 0.0	2.1 ± 0.0	2.1 ± 0.0
% < EAR	3.6 ± 0.4	3.6 ± 0.4	3.5 ± 0.4	4.3 ± 0.6	3.9 ± 0.4	3.9 ± 0.4	3.8 ± 0.4	4.7 ± 0.6
**Niacin ^5^**								
Sample size	10,697	7544	2257	896	10,697	7552	2278	867
Usual Intake	25.8 ± 0.0	25.9 ± 0.0	25.3 ± 0.0	26.1 ± 0.0	25.1 ± 0.0	25.1 ± 0.0	24.6 ± 0.0	25.4 ± 0.0
% < EAR	1.5 ± 0.2	1.4 ± 0.2	1.7 ± 0.2	1.5 ± 0.2	1.6 ± 0.2	1.5 ± 0.2	1.9 ± 0.2	1.6 ± 0.2
% ≥ UL	8.6 ± 0.6	8.4 ± 0.6	9.2 ± 0.8	8.7 ± 0.6	3.6 ± 0.4	3.5 ± 0.4	4.0 ± 0.4	3.7 ± 0.4
**Thiamin ^5,10^**								
Sample size	10,697	7761	2274	662	10,697	7768	2316	613
Usual Intake	1.6 ± 0.0	1.6 ± 0.0	1.6 ± 0.0	1.6 ± 0.0	1.6 ± 0.0	1.6 ± 0.0	1.6 ± 0.0	1.6 ± 0.0
% < EAR	6.7 ± 0.6	6.6 ± 0.4	7.0 ± 0.6	7.2 ± 0.6	7.3 ± 0.6	7.1 ± 0.6	7.7 ± 0.6	7.7 ± 0.6
**Vitamin B_6_^5,9^**								
Sample size	10,697	7541	2067	1089	10,697	7541	2087	1069
Usual Intake	2.1 ± 0.0	2.1 ± 0.0	2.1 ± 0.0	2.1 ± 0.0	2.1 ± 0.0	2.1 ± 0.0	2.0 ± 0.0	2.1 ± 0.0
% < EAR	8.9 ± 0.6	8.6 ± 0.8	10.0 ± 0.6	8.5 ± 0.8	9.6 ± 0.8	9.3 ± 0.8	10.9 ± 0.8	9.2 ± 0.8
**Calcium ^7^**								
Sample size	10,698	7996	2334	368	10,698	7996	2324	378
Usual Intake	995.7 ± 0.4	999.0 ± 0.4	984.7 ± 0.8	994.3 ± 1.8	1021.7 ± 0.4	1024.9 ± 0.4	1011.0 ± 0.8	1020.5 ± 2.0
% < EAR	39.6 ± 1.0	38.7 ± 1.0	42.9 ± 1.0	38.7 ± 1.0	37.6 ± 1.0	36.7 ± 1.0	40.8 ± 1.0	36.8 ± 1.0
% ≥ UL	0.5 ± 0.0	0.5 ± 0.0	0.5 ± 0.0	0.4 ± 0.2	0.7 ± 0.2	0.7 ± 0.2	0.7 ± 0.2	0.6 ± 0.2
**Vitamin C ^8,9^**								
Sample size	10,661	7446	1497	1718	10,661	7446	1487	1728
Usual Intake	85.9 ± 0.0	85.8 ± 0.0	86.7 ± 0.2	85.4 ± 0.2	95.2 ± 0.0	95.1 ± 0.0	96.2 ± 0.2	94.8 ± 0.2
% < EAR	42.6 ± 1.0	42.7 ± 1.0	41.2 ± 1.0	43.0 ± 1.0	37.1 ± 1.0	37.3 ± 1.0	35.9 ± 1.0	37.4 ± 1.0
**Vitamin D ^7,9^**								
Sample size	10,530	2127	6362	2041	10,530	2127	6096	2307
Usual Intake	4.8 ± 0.0	4.8 ± 0.0	4.8 ± 0.0	4.8 ± 0.0	8.4 ± 0.0	8.3 ± 0.0	8.4 ± 0.0	8.4 ± 0.0
% < EAR	96.1 ± 0.6	96.2 ± 0.6	96.1 ± 0.6	96.1 ± 0.6	69.9 ± 1.4	70.6 ± 1.4	69.6 ± 1.4	69.6 ± 1.4

^1^ Data Source: What We Eat in America, NHANES 2009-2012 [22]. ^2^ Usual intake (UI), percent below the Estimated Average Requirement (% < EAR) and percent above or equal to the Tolerable Upper Limit (% ≥ UL) distributions estimated using the National Cancer Institute Method for individuals aged ≥ 2 years, excluding pregnant and lactating women. Accessible via https://epi.grants.cancer.gov/diet/usualintakes/method.html. ^3^ Using Current food label DV and Updated food label DV in Table 1 [17]. ^4^ Percent fortification subgroups for individuals consuming: (a) 0% of DV in the form of fortified food (0%), (b) >0–50% of the DV in the form of fortified food (>0–50% DV), or (c) >50% of DV in the form of fortified food (>50% DV). ^5^ Dietary Reference Intakes for Thiamin, Riboflavin, Niacin, Vitamin B_6_, Folate, Vitamin B_12_, Pantothenic Acid, Biotin, and Choline (1998). ^6^ Dietary Reference Intakes for Vitamin A, Vitamin K, Arsenic, Boron, Chromium, Copper, Iodine, Iron, Manganese, Molybdenum, Nickel, Silicon, Vanadium, and Zinc (2001) [34]. ^7^ Dietary Reference Intakes for Calcium and Vitamin D (2011) [35]. ^8^ Dietary Reference Intakes for Vitamin C, Vitamin E, Selenium, and Carotenoids (2000) [36]. ^9^ Nutrients for which the % ≥ UL = 0 for Current DV and Updated DV models. ^10^ A UL was not available for the following nutrients: Vitamin B_12_, Thiamin, and Riboflavin. DV, Daily Value; US, United States; EAR, Estimated Average Requirement; UI, Usual Intake; UL, Tolerable Upper Limit.

## Abbreviations

CDC: Centers for Disease Control and Prevention; DFE: Dietary Folate Equivalent; DV: Daily Value; DRIs: Dietary Reference Intakes; DS: Dietary supplement; EAR: Estimated Average Requirement; FDA: US Food and Drug Administration; FNB: Food and Nutrition Board; FNDDS: Food and Nutrient Database for Dietary Studies; ILSI, North America: International Life Sciences Institute, North America; ILSI NA-FD: International Life Sciences Institute, North America Fortification Database; IU: International Unit; μg: Micrograms; NCHS: National Center for Health Statistics; NCI: National Cancer Institute; NE: Niacin Equivalent; NHANES: National Health and Nutrition Examination Survey; %DV: Percent of Daily Values; RACC: Reference Amount Customarily Consumed; RAE: Retinal Activity Equivalent; RDA: Recommended Dietary Allowance; SEM: Standard Error of the Mean; UI: Usual Intake; UL: Upper Limit; US: United States; USDA: United States Department of Agriculture; WWEIA: What We Eat in America.

## Appendix A

**Table A1:** Calculations applied to Modified food database to calculate % DV and predict voluntary fortification and nutrient content of foods effective January 2020 and 2021, assuming voluntary fortification levels are adjusted to maintain same % DV as in the Original food database.

Equation Number and Purpose	Equation
1. Calculate % DV contributed per serving based on Current DVs	Old DV% = (Nutrient Total Value ÷ OldDV) × 100
2. Calculate Target Nutrient amount per 100 g using the NewDV	New Total NutrientTarget = (OldDV% × NewDV) × 100
3. Calculate new fortification target (if NewFortValue < 0, set to 0)	New Fort Value = New Total Nutrient − (Intrinsic Value + Enriched Value)

DV, Daily Value.

## Appendix B

Table A2, Table A3, Table A4 and Table A5, which provide comparison modeling data on adult age groups: 19–30 year; 31–50 year; 51–70 year; >70 year.

**Table A2:** Comparison of Current Daily Value (DV) and Updated DV nutrient intake models with overall and percent fortification subgroups for US adults (19–30 years of age) (Mean ± SEM) ^1,2^.

	Current DV ^3^				Updated DV ^3^			
Nutrient	Overall	Percent Fortification ^4^			Overall	Percent Fortification ^4^		
		0%	>0–50%	>50%		0%	>0–50%	>50%
**Vitamin B_12_^5,10^**								
Sample size	2263	1649	309	305	2263	1654	308	301
Usual Intake	5.4 ± 0.0	5.4 ± 0.0	5.4 ± 0.0	5.4 ± 0.0	4.7 ± 0.0	4.6 ± 0.0	4.6 ± 0.0	4.7 ± 0.0
% <EAR	2.4 ± 0.4	2.4 ± 0.4	2.4 ± 0.4	2.4 ± 0.6	3.3 ± 0.6	3.3 ± 0.6	3.3 ± 0.6	3.2 ± 0.6
**Vitamin A ^6,9^**								
Sample size	2264	728	1463	73	2264	981	1221	62
Usual Intake	587.7 ± 0.6	587.4 ± 1.0	587.5 ± 0.6	594.0 ± 3.0	502.6 ± 0.4	503.2 ± 0.6	501.9 ± 0.6	505.6 ± 2.6
% <EAR	52.1 ± 2.8	53.0 ± 3.0	51.5 ± 2.8	55.6 ± 2.6	66.1 ± 2.8	66.3 ± 2.8	65.7 ± 3.0	70.3 ± 2.8
**Zinc ^6,9^**								
Sample size	2266	1844	356	66	2266	1850	351	65
Usual Intake	11.8 ± 0.0	11.8 ± 0.0	11.8 ± 0.0	11.8 ± 0.0	11.5 ± 0.0	11.5 ± 0.0	11.5 ± 0.0	11.4 ± 0.0
% <EAR	18.1 ± 1.4	18.1 ± 1.4	17.0 ± 1.8	21.8 ± 1.4	19.4 ± 1.4	19.4 ± 1.4	18.3 ± 1.8	23.4 ± 1.4
**Riboflavin ^5,10^**								
Sample size	2266	1660	402	204	2266	1660	406	200
Usual Intake	2.1 ± 0.0	2.1 ± 0.0	2.1 ± 0.0	2.1 ± 0.0	2.0 ± 0.0	2.0 ± 0.0	2.0 ± 0.0	2.0 ± 0.0
% <EAR	4.5 ± 0.6	4.5 ± 0.6	4.2 ± 0.6	5.1 ± 0.6	5.0 ± 0.6	5.0 ± 0.6	4.6 ± 0.6	5.6 ± 0.8
**Niacin ^5^**								
Sample size	2266	1575	421	270	2266	1575	430	261
Usual Intake	27.7 ± 0.0	27.7 ± 0.0	27.7 ± 0.0	27.7 ± 0.0	26.8 ± 0.0	26.8 ± 0.0	26.8 ± 0.0	26.9 ± 0.0
% <EAR	0.6 ± 0.2	0.6 ± 0.2	0.6 ± 0.2	0.7 ± 0.2	0.7 ± 0.2	0.7 ± 0.2	0.6 ± 0.2	0.8 ± 0.2
% ≥UL	10.0 ± 1.4	10.0 ± 1.4	10.0 ± 1.4	9.9 ± 1.4	4.3 ± 0.8	4.3 ± 0.8	4.5 ± 0.8	4.4 ± 0.8
**Thiamin ^5,10^**								
Sample size	2266	1685	397	184	2266	1685	409	172
Usual Intake	1.7 ± 0.0	1.7 ± 0.0	1.7 ± 0.0	1.7 ± 0.0	1.6 ± 0.0	1.6 ± 0.0	1.6 ± 0.0	1.6 ± 0.0
% <EAR	5.6 ± 0.8	5.6 ± 0.8	5.3 ± 0.8	6.0 ± 0.8	6.1 ± 0.8	6.1 ± 0.8	5.8 ± 0.8	6.4 ± 1.0
**Vitamin B_6_^5,9^**								
Sample size	2266	1583	380	303	2266	1583	386	297
Usual Intake	2.2 ± 0.0	2.2 ± 0.0	2.2 ± 0.0	2.2 ± 0.0	2.1 ± 0.0	2.1 ± 0.0	2.1 ± 0.0	2.1 ± 0.0
% <EAR	3.7 ± 1.0	3.7 ± 1.0	3.6 ± 1.2	3.6 ± 1.2	4.0 ± 1.0	4.0 ± 1.0	3.9 ± 1.2	4.0 ± 1.0
**Calcium ^7^**								
Sample size	2266	1741	435	90	2266	1741	431	94
Usual Intake	1046.8 ± 0.8	1047.1 ± 0.8	1045.5 ± 1.8	1047.5 ± 4.0	1074.6 ± 0.8	1074.9 ± 1.0	1073.3 ± 1.8	1074.9 ± 4.0
% <EAR	27.3 ± 1.8	27.2 ± 1.8	27.4 ± 1.6	27.3 ± 1.8	25.6 ± 1.8	25.6 ± 1.8	25.7 ± 1.6	26.0 ± 1.6
% ≥UL	0.2 ± 0.0	0.2 ± 0.0	0.2 ± 0.0	0.2 ± 0.2	0.3 ± 0.0	0.3 ± 0.0	0.3 ± 0.0	0.4 ± 0.2
**Vitamin C ^8,9^**								
Sample size	2257	1504	282	471	2257	1504	282	471
Usual Intake	80.0 ± 0.0	80.1 ± 0.2	80.4 ± 0.2	79.8 ± 0.2	90.2 ± 0.2	90.2 ± 0.2	90.7 ± 0.4	89.9 ± 0.2
% <EAR	47.5 ± 2.0	47.5 ± 1.8	46.0 ± 2.0	48.1 ± 2.0	40.7 ± 2.0	40.8 ± 2.0	39.7 ± 2.0	41.3 ± 2.0
**Vitamin D ^7,9^**								
Sample size	2220	563	1191	466	2220	563	1143	514
Usual Intake	4.5 ± 0.0	4.6 ± 0.0	4.5 ± 0.0	4.6 ± 0.0	7.9 ± 0.0	7.9 ± 0.0	7.9 ± 0.0	7.9 ± 0.0
% <EAR	97.1 ± 0.6	97.0 ± 0.6	97.1 ± 0.8	97.0 ± 0.6	73.7 ± 2.4	73.6 ± 2.2	73.8 ± 2.4	73.8 ± 2.4

^1^ Data Source: What We Eat in America, NHANES 2009–2012 [22]. ^2^ Usual intake (UI), percent below the Estimated Average Requirement (% < EAR) and percent above or equal to the Tolerable Upper Limit (% ≥ UL) distributions estimated using the National Cancer Institute Method for individuals aged ≥ 2 years, excluding pregnant and lactating women. Accessible via https://epi.grants.cancer.gov/diet/usualintakes/method.html. ^3^ Using Current food label DV and Updated food label DV in Table 1 [17]. ^4^ Percent fortification subgroups for individuals consuming: (a) 0% of DV in the form of fortified food (0%), (b) >0–50% of the DV in the form of fortified food (>0–50% DV), or (c) >50% of DV in the form of fortified food (>50% DV). ^5^ Dietary Reference Intakes for Thiamin, Riboflavin, Niacin, Vitamin B_6_, Folate, Vitamin B_12_, Pantothenic Acid, Biotin, and Choline (1998). ^6^ Dietary Reference Intakes for Vitamin A, Vitamin K, Arsenic, Boron, Chromium, Copper, Iodine, Iron, Manganese, Molybdenum, Nickel, Silicon, Vanadium, and Zinc (2001) [34]. ^7^ Dietary Reference Intakes for Calcium and Vitamin D (2011) [35]. ^8^ Dietary Reference Intakes for Vitamin C, Vitamin E, Selenium, and Carotenoids (2000) [36]. ^9^ Nutrients for which the % ≥ UL = 0 for Current DV and Updated DV models. ^10^ A UL was not available for the following nutrients: Vitamin B_12_, Thiamin, and Riboflavin. DV, Daily Value; US, United States; EAR, Estimated Average Requirement; UL, Tolerable Upper Limit.

**Table A3:** Comparison of Current Daily Value (DV) and Updated DV nutrient intake models with overall and percent fortification subgroups for US adults (31–50 years of age) ^1,2^ (Mean ± SEM).

	Current DV ^3^				Updated DV ^3^			
Nutrient	Overall	Percent Fortification ^4^			Overall	Percent Fortification ^4^		
		0%	>0–50%	>50%		0%	>0–50%	>50%
**Vitamin B_12_^5,10^**								
Sample size	3522	2693	463	366	3522	2701	458	363
Usual Intake	5.3 ± 0.0	5.3 ± 0.0	5.3 ± 0.0	5.4 ± 0.0	4.7 ± 0.0	4.7 ± 0.0	4.7 ± 0.0	4.7 ± 0.0
% < EAR	2.6 ± 0.4	2.6 ± 0.4	2.7 ± 0.4	2.5 ± 0.4	3.1 ± 0.4	3.1 ± 0.4	3.2 ± 0.6	3.0 ± 0.4
**Vitamin A ^6,9^**								
Sample size	3518	986	2422	110	3518	1364	2069	85
Usual Intake	631.7 ± 0.4	631.1 ± 0.8	631.9 ± 0.6	632.9 ± 2.6	540.1 ± 0.4	539.6 ± 0.6	540.3 ± 0.4	553.1 ± 1.4
% < EAR	45.1 ± 2.4	46.2 ± 2.6	44.6 ± 2.4	47.1 ± 2.4	59.1 ± 2.4	60.1 ± 2.4	58.4 ± 2.4	60.5 ± 2.2
**Zinc ^6,9^**								
Sample size	3526	2931	519	76	3526	2949	506	71
Usual Intake	12.1 ± 0.0	12.1 ± 0.0	12.1 ± 0.0	12.2 ± 0.0	11.9 ± 0.0	11.9 ± 0.0	11.9 ± 0.0	11.9 ± 0.0
% < EAR	15.9 ± 1.0	15.8 ± 1.0	15.8 ± 1.0	17.8 ± 1.6	16.6 ± 1.2	16.6 ± 1.2	16.5 ± 1.2	18.9 ± 1.6
**Riboflavin ^5,10^**								
Sample size	3526	2669	602	255	3526	2670	606	250
Usual Intake	2.2 ± 0.0	2.2 ± 0.0	2.2 ± 0.0	2.2 ± 0.0	2.1 ± 0.0	2.1 ± 0.0	2.1 ± 0.0	2.1 ± 0.0
% < EAR	3.1 ± 0.4	3.2 ± 0.4	2.8 ± 0.4	3.6 ± 0.6	3.3 ± 0.6	3.4 ± 0.6	3.0 ± 0.4	3.9 ± 0.6
**Niacin ^5^**								
Sample size	3526	2584	631	311	3526	2586	638	302
Usual Intake	27.0 ± 0.0	26.9 ± 0.0	27.0 ± 0.0	27.0 ± 0.0	26.3 ± 0.0	26.3 ± 0.0	26.3 ± 0.0	26.3 ± 0.0
% < EAR	0.8 ± 0.2	0.8 ± 0.2	0.8 ± 0.2	0.8 ± 0.2	0.8 ± 0.2	0.8 ± 0.2	0.8 ± 0.2	0.9 ± 0.2
% ≥ UL	6.5 ± 0.6	6.5 ± 0.6	6.5 ± 0.8	6.5 ± 0.6	2.6 ± 0.4	2.6 ± 0.4	2.6 ± 0.4	2.6 ± 0.4
**Thiamin ^5,10^**								
Sample size	3526	2677	644	205	3526	2680	654	192
Usual Intake	1.7 ± 0.0	1.7 ± 0.0	1.7 ± 0.0	1.7 ± 0.0	1.6 ± 0.0	1.6 ± 0.0	1.6 ± 0.0	1.6 ± 0.0
% < EAR	5.5 ± 0.6	5.5 ± 0.6	5.2 ± 0.6	5.5 ± 0.6	5.8 ± 0.6	5.9 ± 0.6	5.5 ± 0.6	6.0 ± 0.6
**Vitamin B_6_^5,9^**								
Sample size	3526	2577	586	363	3526	2577	590	359
Usual Intake	2.2 ± 0.0	2.2 ± 0.0	2.2 ± 0.0	2.2 ± 0.0	2.1 ± 0.0	2.1 ± 0.0	2.1 ± 0.0	2.1 ± 0.0
% < EAR	3.7 ± 0.6	3.7 ± 0.6	3.7 ± 0.6	3.7 ± 0.6	3.8 ± 0.6	3.8 ± 0.6	3.8 ± 0.6	3.9 ± 0.6
**Calcium ^7^**								
Sample size	3527	2760	655	112	3527	2760	653	114
Usual Intake	1032.4 ± 0.6	1032.7 ± 0.8	1031.5 ± 1.4	1030.5 ± 3.4	1056.2 ± 0.6	1056.5 ± 0.8	1055.3 ± 1.6	1054.5 ± 3.6
% < EAR	28.6 ± 1.8	28.6 ± 1.8	28.7 ± 1.8	28.4 ± 1.6	27.2 ± 1.6	27.2 ± 1.6	27.3 ± 1.6	27.0 ± 1.4
% ≥ UL	0.2 ± 0.0	0.2 ± 0.0	0.2 ± 0.0	0.1 ± 0.0	0.3 ± 0.0	0.3 ± 0.0	0.3 ± 0.0	0.3 ± 0.0
**Vitamin C ^8,9^**								
Sample size	3518	2561	417	540	3518	2561	412	545
Usual Intake	81.9 ± 0.0	81.9 ± 0.0	81.6 ± 0.2	82.1 ± 0.2	90.5 ± 0.0	90.5 ± 0.2	90.2 ± 0.2	90.7 ± 0.2
% < EAR	45.6 ± 1.6	45.8 ± 1.6	45.0 ± 1.8	45.4 ± 1.6	40.2 ± 1.4	40.4 ± 1.4	39.4 ± 1.4	40.0 ± 1.4
**Vitamin D ^7,9^**								
Sample size	3474	764	2012	698	3474	764	1925	785
Usual Intake	4.7 ± 0.0	4.7 ± 0.0	4.7 ± 0.0	4.7 ± 0.0	8.1 ± 0.0	8.1 ± 0.0	8.1 ± 0.0	8.1 ± 0.0
% < EAR	96.5 ± 0.8	96.5 ± 0.8	96.5 ± 0.6	96.6 ± 0.8	71.9 ± 2.2	71.9 ± 2.4	71.9 ± 2.2	71.9 ± 2.2

^1^ Data Source: What We Eat in America, NHANES 2009–2012 [22]. ^2^ Usual intake (UI), percent below the Estimated Average Requirement (% < EAR) and percent above or equal to the Tolerable Upper Limit (% ≥ UL) distributions estimated using the National Cancer Institute Method for individuals aged ≥2 years, excluding pregnant and lactating women. Accessible via https://epi.grants.cancer.gov/diet/usualintakes/method.html. ^3^ Using Current food label DV and Updated food label DV in Table 1 [17]. ^4^ Percent fortification subgroups for individuals consuming: (a) 0% of DV in the form of fortified food (0%), (b) >0–50% of the DV in the form of fortified food (>0–50% DV), or (c) >50% of DV in the form of fortified food (>50% DV). ^5^ Dietary Reference Intakes for Thiamin, Riboflavin, Niacin, Vitamin B_6_, Folate, Vitamin B_12_, Pantothenic Acid, Biotin, and Choline (1998) ^6^ Dietary Reference Intakes for Vitamin A, Vitamin K, Arsenic, Boron, Chromium, Copper, Iodine, Iron, Manganese, Molybdenum, Nickel, Silicon, Vanadium, and Zinc (2001) [34].^7^ Dietary Reference Intakes for Calcium and Vitamin D (2011) [35]. ^8^ Dietary Reference Intakes for Vitamin C, Vitamin E, Selenium, and Carotenoids (2000) [36]. ^9^ Nutrients for which the % ≥ UL = 0 for Current DV and Updated DV models. ^10^ A UL was not available for the following nutrients: vitamin B_12_, Thiamin, and Riboflavin. DV, Daily Value; US, United States; EAR, Estimated Average Requirement; UL, Tolerable Upper Limit.

**Table A4:** Comparison of Current Daily Value (DV) and Updated DV nutrient intake models with overall and percent fortification subgroups for US adults (51–70 years of age) (Mean ± SEM) ^1,2^.

	Current DV ^3^				Updated DV ^3^			
Nutrient	Overall	Percent Fortification ^4^			Overall	Percent Fortification ^4^		
		0%	>0–50%	>50%		0%	>0–50%	>50%
**Vitamin B_12_^5,10^**								
Sample size	3319	2528	511	280	3319	2532	510	277
Usual Intake	5.2 ± 0.0	5.2 ± 0.0	5.2 ± 0.0	5.2 ± 0.0	4.5 ± 0.0	4.5 ± 0.0	4.5 ± 0.0	4.5 ± 0.0
% < EAR	3.0 ± 0.6	3.0 ± 0.6	3.0 ± 0.6	3.1 ± 0.6	3.8 ± 0.6	3.8 ± 0.6	3.8 ± 0.6	3.8 ± 0.6
**Vitamin A ^6,9^**								
Sample size	3326	746	2496	84	3326	1097	2164	65
Usual Intake	686.7 ± 0.4	688.8 ± 1.0	686.1 ± 0.6	684.1 ± 3.2	585.0 ± 0.4	586.5 ± 0.8	584.3 ± 0.6	583.4 ± 3.0
% < EAR	37.7 ± 3.0	38.2 ± 2.8	37.4 ± 3.0	41.4 ± 3.6	51.8 ± 3.6	52.4 ± 3.4	51.3 ± 3.6	56.3 ± 4.0
**Zinc ^6,9^**								
Sample size	3330	2761	512	57	3330	2776	503	51
Usual Intake	11.5 ± 0.0	11.5 ± 0.0	11.5 ± 0.0	11.5 ± 0.0	11.2 ± 0.0	11.2 ± 0.0	11.2 ± 0.0	11.2 ± 0.0
% < EAR	20.1 ± 1.6	20.3 ± 1.6	19.2 ± 1.6	22.1 ± 2.0	21.3 ± 1.6	21.4 ± 1.6	20.2 ± 1.6	22.3 ± 2.0
**Riboflavin ^5,10^**								
Sample size	3330	2482	701	147	3330	2488	667	175
Usual Intake	2.2 ± 0.0	2.2 ± 0.0	2.2 ± 0.0	2.2 ± 0.0	2.1 ± 0.0	2.1 ± 0.0	2.1 ± 0.0	2.1 ± 0.0
% < EAR	3.0 ± 0.4	3.0 ± 0.4	2.9 ± 0.4	3.3 ± 0.4	3.2 ± 0.4	3.2 ± 0.4	3.0 ± 0.6	3.4 ± 0.6
**Niacin ^5^**								
Sample size	3330	2459	672	199	3330	2463	674	193
Usual Intake	25.1 ± 0.0	25.1 ± 0.0	25.1 ± 0.0	25.1 ± 0.0	24.5 ± 0.0	24.5 ± 0.0	24.5 ± 0.0	24.4 ± 0.0
% < EAR	1.5 ± 0.2	1.5 ± 0.2	1.4 ± 0.2	1.6 ± 0.2	1.6 ± 0.2	1.6 ± 0.2	1.5 ± 0.2	1.7 ± 0.2
% ≥ UL	7.2 ± 1.0	7.2 ± 1.0	7.3 ± 1.0	7.3 ± 1.0	2.9 ± 0.6	2.9 ± 0.6	2.9 ± 0.4	2.9 ± 0.6
**Thiamin ^5,10^**								
Sample size	3330	2479	691	160	3330	2482	701	147
Usual Intake	1.6 ± 0.0	1.6 ± 0.0	1.6 ± 0.0	1.6 ± 0.0	1.6 ± 0.0	1.6 ± 0.0	1.6 ± 0.0	1.6 ± 0.0
% < EAR	6.8 ± 0.8	6.8 ± 0.8	6.6 ± 0.8	7.2 ± 1.0	7.3 ± 0.8	7.3 ± 0.8	7.2 ± 0.8	7.6 ± 1.0
**Vitamin B_6_^5,9^**								
Sample size	3330	2455	619	256	3330	2455	626	249
Usual Intake	2.1 ± 0.0	2.1 ± 0.0	2.1 ± 0.0	2.1 ± 0.0	2.0 ± 0.0	2.0 ± 0.0	2.0 ± 0.0	2.0 ± 0.0
% < EAR	13.2 ± 1.0	13.1 ± 1.2	13.0 ± 1.0	13.8 ± 1.2	14.1 ± 1.2	14.0 ± 1.2	13.9 ± 1.2	14.6 ± 1.2
**Calcium ^7^**								
Sample size	3330	2483	736	111	3330	2483	733	114
Usual Intake	972 ± 0.6	972 ± 0.8	972.7 ± 1.4	966.8 ± 3.2	997.1 ± 0.6	997.1 ± 0.8	997.9 ± 1.4	991.3 ± 3.4
% < EAR	46.5 ± 2.4	46.1 ± 2.4	47.8 ± 2.4	44.3 ± 2.2	44.2 ± 2.4	43.9 ± 2.4	45.5 ± 2.2	42.3 ± 2.2
% ≥ UL	1.0 ± 0.2	1.0 ± 0.2	1.0 ± 0.2	0.8 ± 0.4	1.4 ± 0.2	1.3 ± 0.2	1.4 ± 0.4	1.2 ± 0.4
**Vitamin C ^8,9^**								
Sample size	3315	2392	461	462	3315	2392	458	465
Usual Intake	90.8 ± 0.0	90.8 ± 0.2	90.7 ± 0.2	90.8 ± 0.2	100.0 ± 0.2	100.0 ± 0.2	100.0 ± 0.2	100.1 ± 0.2
% < EAR	38.7 ± 1.8	38.9 ± 1.8	38.1 ± 2.0	38.4 ± 1.8	33.9 ± 1.6	34.1 ± 1.6	33.2 ± 1.8	33.7 ± 1.6
**Vitamin D ^7,9^**								
Sample size	3273	624	2104	545	3273	624	2010	639
Usual Intake	4.9 ± 0.0	4.9 ± 0.0	4.9 ± 0.0	4.9 ± 0.0	8.5 ± 0.0	8.5 ± 0.0	8.5 ± 0.0	8.5 ± 0.0
% < EAR	95.9 ± 0.6	95.9 ± 0.6	96.0 ± 0.8	95.9 ± 0.6	69.3 ± 1.8	69.1 ± 1.6	69.3 ± 1.8	69.3 ± 1.8

^1^ Data Source: What We Eat in America, NHANES 2009–2012 [22]. ^2^ Usual intake (UI), percent below the Estimated Average Requirement (% < EAR) and percent above or equal to the Tolerable Upper Limit (% ≥ UL) distributions estimated using the National Cancer Institute Method for individuals aged ≥2 years, excluding pregnant and lactating women. Accessible via https://epi.grants.cancer.gov/diet/usualintakes/method.html. ^3^ Using Current food label DV and Updated food label DV in Table 1 [17]. ^4^ Percent fortification subgroups for individuals consuming: (a) 0% of DV in the form of fortified food (0%), (b) >0–50% of the DV in the form of fortified food (>0–50% DV), or (c) >50% of DV in the form of fortified food (>50% DV). ^5^ Dietary Reference Intakes for Thiamin, Riboflavin, Niacin, Vitamin B_6_, Folate, Vitamin B_12_, Pantothenic Acid, Biotin, and Choline (1998) ^6^ Dietary Reference Intakes for Vitamin A, Vitamin K, Arsenic, Boron, Chromium, Copper, Iodine, Iron, Manganese, Molybdenum, Nickel, Silicon, Vanadium, and Zinc (2001) [34].^7^ Dietary Reference Intakes for Calcium and Vitamin D (2011) [35]. ^8^ Dietary Reference Intakes for Vitamin C, Vitamin E, Selenium, and Carotenoids (2000) [36]. ^9^ Nutrients for which the % ≥ UL = 0 for Current DV and Updated DV models. ^10^ A UL was not available for the following nutrients: Vitamin B_12_, Thiamin, and Riboflavin. DV, Daily Value; US, United States; EAR, Estimated Average Requirement; UL, Tolerable Upper Limit.

**Table A5:** Comparison of Current Daily Value (DV) and Updated DV nutrient intake models with overall and percent fortification subgroups for US adults (> 70 years of age) (Mean ± SEM) ^1,2^.

	Current DV ^3^				Updated DV ^3^			
Nutrient	Overall	Percent Fortification ^4^			Overall	Percent Fortification ^4^		
		0%	>0–50%	>50%		0%	>0–50%	>50%
**Vitamin B_12_^5,10^**								
Sample size	1573	993	391	189	1573	996	392	185
Usual Intake	5.1 ± 0.0	5.1 ± 0.0	5.1 ± 0.0	5.1 ± 0.0	4.4 ± 0.0	4.4 ± 0.0	4.4 ± 0.0	4.4 ± 0.0
% < EAR	3.3 ± 0.6	3.2 ± 0.6	3.4 ± 0.8	3.4 ± 0.8	4.8 ± 1.0	4.7 ± 1.0	4.9 ± 1.2	5.2 ± 1.6
**Vitamin A ^6,9^**								
Sample size	1573	201	1300	72	1573	305	1210	58
Usual Intake	710.6 ± 0.8	711.2 ± 2.0	710.5 ± 0.8	709.3 ± 3.4	585.6 ± 0.6	585.6 ± 1.4	585.6 ± 0.6	586.1 ± 3.2
% < EAR	34.3 ± 2.2	35.2 ± 2.2	34.0 ± 2.2	36.9 ± 2.4	51.4 ± 2.2	51.9 ± 2.4	51.1 ± 2.2	53.7 ± 2.4
**Zinc ^6,9^**								
Sample size	1575	1130	386	59	1575	1141	378	56
Usual Intake	10.4 ± 0.0	10.4 ± 0.0	10.4 ± 0.0	10.4 ± 0.0	10.1 ± 0.0	10.1 ± 0.0	10.1 ± 0.0	10.1 ± 0.0
% < EAR	27.9 ± 1.6	28.0 ± 1.6	27.6 ± 1.6	29.8 ± 2	30.6 ± 1.6	30.5 ± 1.6	30.6 ± 1.6	32.7 ± 2.0
**Riboflavin ^5,10^**								
Sample size	1575	927	520	128	1575	928	522	125
Usual Intake	2.0 ± 0.0	2.0 ± 0.0	2.0 ± 0.0	2.0 ± 0.0	2.0 ± 0.0	2.0 ± 0.0	2.0 ± 0.0	2.0 ± 0.0
% < EAR	4.7 ± 0.6	4.6 ± 0.6	4.7 ± 0.6	5.8 ± 1.4	5.3 ± 0.6	5.2 ± 0.6	5.3 ± 0.6	6.5 ± 1.6
**Niacin ^5^**								
Sample size	1575	926	533	116	1575	928	536	111
Usual Intake	21.8 ± 0.0	21.8 ± 0.0	21.8 ± 0.0	21.7 ± 0.0	21.1 ± 0.0	21.1 ± 0.0	21.1 ± 0.0	21.0 ± 0.0
% < EAR	4.2 ± 0.4	4.1 ± 0.4	4.2 ± 0.6	5.0 ± 1.0	4.8 ± 0.6	4.6 ± 0.6	4.8 ± 0.6	5.6 ± 1.2
% ≥ UL	14.2 ± 1.6	14.2 ± 1.6	14.3 ± 1.6	14.1 ± 1.8	6.5 ± 1.0	6.5 ± 1.0	6.6 ± 1.0	6.2 ± 1.2
**Thiamin ^5,10^**								
Sample size	1575	920	542	113	1575	921	552	102
Usual Intake	1.5 ± 0.0	1.5 ± 0.0	1.5 ± 0.0	1.5 ± 0.0	1.4 ± 0.0	1.4 ± 0.0	1.4 ± 0.0	1.5 ± 0.0
% < EAR	10.9 ± 1.0	10.8 ± 1.0	10.9 ± 1.0	11.9 ± 1.6	12.4 ± 1.0	12.3 ± 1.0	12.4 ± 1.0	13.2 ± 1.6
**Vitamin B_6_^5,9^**								
Sample size	1575	926	482	167	1575	926	485	164
Usual Intake	1.9 ± 0.0	1.9 ± 0.0	1.9 ± 0.0	1.9 ± 0.0	1.9 ± 0.0	1.9 ± 0.0	1.9 ± 0.0	1.9 ± 0.0
% < EAR	19.1 ± 1.4	18.9 ± 1.4	19.1 ± 1.4	19.8 ± 1.8	21.0 ± 1.6	20.9 ± 1.6	21.0 ± 1.6	21.8 ± 1.8
**Calcium ^7^**								
Sample size	1575	1012	508	55	1575	1012	507	56
Usual Intake	890.0 ± 0.8	890.3 ± 1.0	889.5 ± 1.4	889.1 ± 4.4	920.6 ± 0.8	920.9 ± 1.0	920.1 ± 1.6	919.5 ± 4.6
% < EAR	67.5 ± 1.4	67.4 ± 1.4	67.6 ± 1.4	67.0 ± 1.8	64.0 ± 1.6	64.0 ± 1.6	64.1 ± 1.6	63.5 ± 1.6
% ≥ UL	0.5 ± 0.0	0.5 ± 0.0	0.5 ± 0.0	0.5 ± 0.2	0.8 ± 0.2	0.8 ± 0.2	0.8 ± 0.2	0.7 ± 0.2
**Vitamin C ^8,9^**								
Sample size	1571	989	337	245	1571	989	335	247
Usual Intake	92.8 ± 0.2	92.6 ± 0.2	93.0 ± 0.2	93.1 ± 0.4	102.8 ± 0.2	102.6 ± 0.2	103.0 ± 0.4	103.2 ± 0.4
% < EAR	36.8 ± 1.8	36.9 ± 1.8	36.8 ± 1.8	36.4 ± 1.8	31.9 ± 1.6	32.0 ± 1.6	32.0 ± 1.6	31.4 ± 1.8
**Vitamin D ^7,9^**								
Sample size	1563	176	1055	332	1563	176	1018	369
Usual Intake	5.3 ± 0.0	5.4 ± 0.0	5.3 ± 0.0	5.3 ± 0.0	9.5 ± 0.0	9.5 ± 0.0	9.5 ± 0.0	9.5 ± 0.0
% < EAR	94.1 ± 0.8	94.0 ± 0.8	94.1 ± 1.0	94.0 ± 0.8	61.2 ± 2.0	61.1 ± 2.0	61.2 ± 2.0	61.3 ± 2.0

^1^ Data Source: What We Eat in America, NHANES 2009–2012 [22]. ^2^ Usual intake (UI), percent below the Estimated Average Requirement (% < EAR) and percent above or equal to the Tolerable Upper Limit (% ≥ UL) distributions estimated using the National Cancer Institute Method for individuals aged ≥2 years, excluding pregnant and lactating women. Accessible via https://epi.grants.cancer.gov/diet/usualintakes/method.html. ^3^ Using Current food label DV and Updated food label DV in Table 1 [17]. ^4^ Percent fortification subgroups for individuals consuming: (a) 0% of DV in the form of fortified food (0%), (b) >0–50% of the DV in the form of fortified food (>0–50% DV), or (c) >50% of DV in the form of fortified food (>50% DV). ^5^ Dietary Reference Intakes for Thiamin, Riboflavin, Niacin, Vitamin B_6_, Folate, Vitamin B_12_, Pantothenic Acid, Biotin, and Choline (1998) ^6^ Dietary Reference Intakes for Vitamin A, Vitamin K, Arsenic, Boron, Chromium, Copper, Iodine, Iron, Manganese, Molybdenum, Nickel, Silicon, Vanadium, and Zinc (2001) [34].^7^ Dietary Reference Intakes for Calcium and Vitamin D (2011) [35]. ^8^ Dietary Reference Intakes for Vitamin C, Vitamin E, Selenium, and Carotenoids (2000) [36]. ^9^ Nutrients for which the % ≥ UL = 0 for Current DV and Updated DV models. ^10^ A UL was not available for the following nutrients: Vitamin B_12_, Thiamin, and Riboflavin. DV, Daily Value; US, United States; EAR, Estimated Average Requirement; UL, Tolerable Upper Limit.
